# Effects of acoustic levitation on the development of zebrafish, *Danio rerio*, embryos

**DOI:** 10.1038/srep13596

**Published:** 2015-09-04

**Authors:** Maria Sundvik, Heikki J. Nieminen, Ari Salmi, Pertti Panula, Edward Hæggström

**Affiliations:** 1 Neuroscience Center and Department of Anatomy, POB 63, 00014 University of Helsinki, Finland; 2 Electronics Research Laboratory and Department of Physics, POB 64, 00014 University of Helsinki, Finland

## Abstract

Acoustic levitation provides potential to characterize and manipulate material such as solid particles and fluid in a wall-less environment. While attempts to levitate small animals have been made, the biological effects of such levitation have been scarcely documented. Here, our goal was to explore if zebrafish embryos can be levitated (peak pressures at the pressure node and anti-node: 135 dB and 144 dB, respectively) with no effects on early development. We levitated the embryos (*n *= 94) at 2–14 hours post fertilization (hpf) for 1000 (*n *= 47) or 2000 seconds (*n *= 47). We compared the size and number of trunk neuromasts and otoliths in sonicated samples to controls (*n *= 94), and found no statistically significant differences (*p *> 0.05). While mortality rate was lower in the control group (22.3%) compared to that in the 1000 s (34.0%) and 2000 s (42.6%) levitation groups, the differences were statistically insignificant (*p *> 0.05). The results suggest that acoustic levitation for less than 2000 sec does not interfere with the development of zebrafish embryos, but may affect mortality rate. Acoustic levitation could potentially be used as a non-contacting wall-less platform for characterizing and manipulating vertebrae embryos without causing major adverse effects to their development.

Acoustic levitation (AL) is an established actuation technique that can trap^1^,^2^, translate^3^, and rotate^4^ material, *e.g.* particles, droplets or cells, in fluids (air or liquid) without physically touching the sample. In practice, AL provides a wall-less non-touching platform to run experiments on small samples^5^,^6^. When the vertical acoustic forces interacting with the sample (forces that point upwards and downwards around the pressure node of the standing wave) balance the gravitation force, the sample is vertically trapped (Fig. 1). This permits one to (i) study or (ii) manipulate the sample with good spatial access (*i.e.* no sample holder obstructing the access), (iii) reduce the risk for artifacts arising from contamination from the sample holder/container (chemical/bacterial/viral), and (iv) avoid inducing shear forces arising from conventional mechanical support that the sample would be in contact with.

Studies exist on levitating living cells such as such as murine embryonic stem cells^7^, blood cells^8^, and small animals such as lady bug, ant, and adult fish^6^. There is concern with potential adverse effects introduced by AL. While articles report on the biological effects of levitation on cells^7^,^8^,^9^, there are few systematic studies on the biological effects (*e.g.* development or vitality) of AL on small vertebrae. One study reported, based on qualitative analysis, that the vitality of juvenile fish was affected by the levitation^6^. However, the authors speculated that this finding potentially was due to lack of water immersion of the fish during the levitation. While the ant and ladybug were “still with sufficient vitality” after 30 min of levitation, no negative effects on the vitality of other small animals such as beetle, spider, or bee by AL were reported^6^. To date, the adverse effects of AL on small animals, therefore, remains mostly unknown. Since AL produces mechanical pressure fluctuations of air in the kHz range equivalent to >135 dB sound pressure level (SPL), the AL could potentially affect the biology of the mechanically sensitive organs, *e.g.* hearing or pressure sensing/sensitive organs.

The zebrafish has extensively been used as a small animal model organism in biomedical research^10^,^11^,^12^. During the first days of its lifespan it is transparent and exhibits a rapid *ex utero* development, which allows easy visualization of organ development and genetic manipulation^13^,^14^. The major organs are visible 24 hours post fertilization (hpf) and by 5 days post fertilization (dpf) the zebrafish exhibits an array of measurable behaviors^15^,^16^,^17^,^18^,^19^,^20^. The zebrafish use e.g. the lateral line and inner ear to receive information about the outside world. These two systems exhibit anatomical and functional similarities related to signal transduction: they both carry sensory hair cells.

The sensory hair cells and otoliths of the inner ear respond to sound, gravity, and acceleration^21^. Zebrafish otoliths become visible at somite stage #21 (19–19.5 hpf) and increase in diameter over the next hours. At 24 hpf the otoliths are 7–8 μm in diameter, and the first sensory hair cells attached to the otolith can be detected^21^. The major inner ear structures have developed and are clearly seen at 5 dpf; each crista consists of approximately 20 hair cells whereas the two maculae contain 50–60 hair cells. The posterior otolith is 55 μm in diameter and the anterior otolith is 45 μm in diameter^21^. At this age the ear is fully functional, and the animals can respond to acoustic startle and gravity^22^.

The lateral line system in zebrafish carries only mechanosensory organs, *i.e.* neuromasts. One neuromast comprises supporting cells surrounding a center with 15–20 mechanosensory hair cells that respond to water flow^23^,^24^. The posterior lateral line originates posterior of the otic vesicle and delaminates around 20 hpf to form an anterior and posterior compartment. The posterior lateral line is formed by a moving primodium that deposits 7 clusters of cells, the proneuromasts, which matures into neuromasts within hours. When the primodium reaches the tail, it fragments and forms two to three terminal neuromasts^23^. The hair cells in the neuromasts are not assigned an auditory function in contrast to the hair cells of the ear^23^. Early studies on mutations that have been shown to affect the morphogenesis of the ear have not been associated with disturbed lateral line formation^25^. This suggests that the development, differentiation, and formation of these two systems are different. However, recent results indicate that the gene *miles-apart* is required for formation of both otoliths and hair cells in zebrafish^26^. Overexpression of *miles-apart* induces an overexpression of neuromasts in the posterior lateral line^26^.

In this study, we investigated whether AL affects the development and vitality of zebrafish embryos. Zebrafish has during last decade been extensively used as a model organism, and many approaches to manipulate developing zebrafish embryo are available^13^,^14^,^15^. We chose to study a fish species because previous literature suggests that levitation may affect the vitality of juvenile fish and to provide a new approach for handling zebrafish in research. We conducted the study on zebrafish embryos since we believe that external influence is most likely to adversely affect the development of young fish. We studied the development of the posterior lateral line neuromasts and the otoliths of the inner ear as these are specifically important for processing mechanical and acoustic stimuli, respectively.

## Results

In total, 94 embryos were collected into the control group and 47 and 47 embryos were levitated in the 1000 s and 2000 s groups, respectively (Fig. 2). At five dpf, 73 (21 dead, *i.e.* 22.3%), 31 (16 dead, *i.e.* 34.0%) and 27 (20 dead, *i.e.* 42.6%) embryos were alive in the control, 1000 s and 2000 s groups, respectively. These data are merged from three individual experiments. No significant differences were observed in the death rate between the three different groups (One-way analysis of variance, *p *> 0.05, followed by Tukey’s Multiple Comparison Test, see Table 1 for details).

The number of posterior lateral line neuromasts in 5 dpf old zebrafish larvae that were levitated at 1.5–14 hpf were not altered when compared with control animals (Fig. 3). Neither in the group levitated for 1000 s nor in the group levitated for 2000 s could a significant difference be detected when compared with the control group (One-way analysis of variance, *p *> 0.05, followed by Tukey’s Multiple Comparison Test, see Table 2 for details). Neither was the morphology of the posterior lateral line neuromasts altered in the same animals (Fig. 3). The fish were levitated at 1.5–14 hpf, and the main mortality of untreated zebrafish occurred during the first 24 hours of development.

We also assessed whether the otolith development was abnormal in the levitated zebrafish larvae when compared with control animals. Both otoliths were present in the inner ear of the levitated fish, and the morphology of the otoliths was unaltered by levitation (Fig. 4). Both the anterior and the posterior otolith were clearly visible, and we detected no apparent change in the size or location of the otoliths relative to each other. The diameter of the anterior and posterior otoliths were measured and no statistical difference was detected when the levitated animals were compared with control animals (Student’s t-test, *p > *0.05).

**Figure 1:**
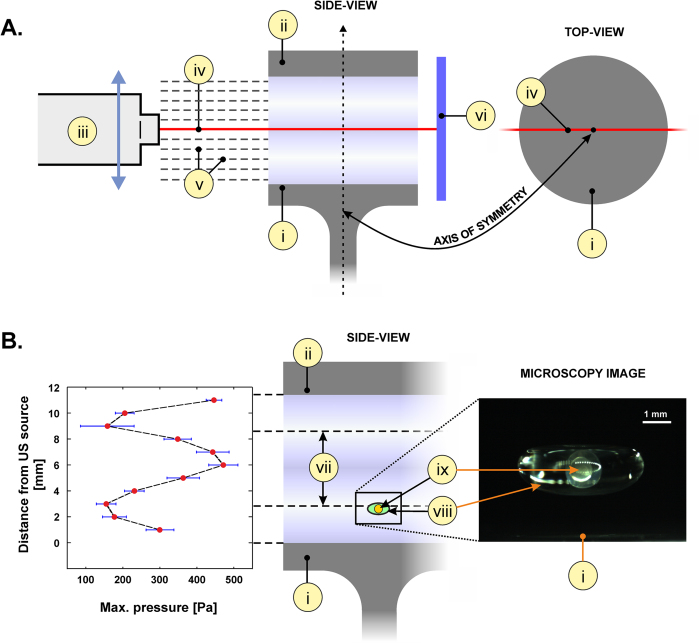
(**A**). Measurement setup to characterize the ultrasound field: (i) Langevin (ultrasound) transducer, (ii) metallic reflector, (iii) laser Doppler vibrometer (*i.e.* LDV), (iv) laser beam, (v) vertical positions of the laser beam (dashed lines) and (vi) glass plate that reflects the laser beam back to the LDV. As the LDV is moved vertically, the sound field in the AL can be determined. (**B**): Maximum spatial average pressure through the levitator (left) as measured with the setup demonstrated in Fig. 1A. Dashed lines (right, vii) represent the pressure nodes, (viii) is the E3 medium droplet in which the levitated embryo (ix) is immersed. Microscopy image (right) demonstrates a levitated embryo.

Based on the optical measurements, the magnitude of the maximum pressure (averaged spatially along the radius of the levitator) was greatest, *i.e.* 472 Pa, at the pressure anti-node and smallest, *i.e.* 155 Pa, at the pressure node (Fig. 1B). The location (measurement accuracy 0.5 mm) of pressure nodes corresponded to locations expected, *i.e.* distances λ/4 (2.5 mm) and 3λ/4 (7.4 mm) above the Langevin transducer. The location of the pressure node closer to the transducer, approximately corresponds to the location of the levitated embryo.

Safety assessment revealed that 100% mortality rate was achieved when (i) the embryo spontaneously exited the levitator (Table 3) or (ii) E3 medium droplet and the embryo had dried and the embryo was manually extracted from the levitator (Table 3). The time points where sample (*i.e.* E3 medium droplet + embryo) height and volume reached embryo height and volume, respectively, are also summarized in Table 3.

## Discussions

The goal of this study was to investigate if vitality and development of zebrafish are affected by AL. We observed no statistically significant difference in the number of dead larvae 5 dpf in the levitation groups as compared to the control group. The morphology of the pressure and sound sensitive otholits and neuromasts developed normally up to 5 dpf. The results, therefore, suggest that AL does not induce visually detectable harm the development of zebrafish larvae in regard to organs that could be expected to be damaged by high sound pressure levels.

The zebrafish has high regeneration capacity and has therefore been used in studies of regeneration^27^. Also the hair cells of the lateral line regenerate after CuSO_4_^24^,^28^ and neomycin treatment^29^. At 42 hpf the neurogenesis from the ear epithelium has ended^21^. All levitations were done before the animals reached an age of 14 hpf at the 6-somite stage. This is well before the neuromasts and otoliths have formed, as they form at 19 and 20 hpf, respectively. The development of the posterior lateral line has been divided into two different stages; one before 20 hpf when the primodium forms, and a later stage beyond 20 hpf when the primodum travels towards the tip of the tail^30^. Our findings do not reveal whether AL induced no harm to the developing primodium or whether any eventual damage caused by levitation was already rescued before we assessed it. The fish were left to develop until 5 dpf as we were interested in determining whether the AL was harmful *per se* for the development; we did not investigate instant effects.

A study on the effect of histone deacetylase (HDAC) activity on the development of the posterior lateral line^30^ showed that inhibiting HDAC activity between 6–14 hpf reduced the number of neuromasts in the treated animals when compared with wild-type untreated animals. Treatment with the same HDAC inhibitor during a later developmental period, from 14 to 20 hpf did not alter the number of neuromasts^30^. These two findings combined indicate that the stage between 6 and 14 hpf appears to be essential for the formation of the initial primodium. Comparing these findings with our results suggests that the levitation is not harmful to the developing primodium. The migration of the posterior lateral line primodium requires presence of the Gβ1 subunit that is suggested to act *via* rearranging the actin cytoskeleton in the leading region of cells.

The results related to the vitality of zebrafish embryos are somewhat contradictory to a previous study^6^, which reported that the vitality of levitated fish was compromised. The explanation proposed by those authors was that the vitality of the fish was affected by inadequate water supply. The present study confirms that zebrafish embryos, immersed in E3 medium during the levitation event, did not die immediately after AL. Moreover, their vitality was observed to be normal at 5 dpf. Therefore, there is currently no existing data to suggest that AL would be harmful to small vertebrates. It is notable that evaporation from the E3 medium droplet surrounding the embryo occurs during the levitation and may explain the minor, but statistically insignificant mortality rate^31^. Therefore, levitation exceeding 2000 s may need to consider ways to avoid evaporation. Mortality rate of 100% was achieved at levitation times 3428–5578 s (*n *= 11) representing the ultimate time limit for AL experiments described in this study.

Sound and pressure sensitive organs were selected for this investigation, because the intense sound pressure in the levitator, *i.e.* 155–472 Pa (135–144 dB SPL) at 35.2 kHz, was considered sufficiently high to potentially damage these organs. We observed no developmental effects induced by AL in sound and pressure sensitive organs. However, the results are promising and propose that research towards exploiting AL as a non-contacting and a non-destructive test platform for zebrafish embryos and, potentially fish, should be continued.

In addition to AL, there are other levitation techniques that rely on physical fields such as light^32^, electrostatic^33^ and magnetic^34^ fields. Common to all levitation techniques is the goal to trap matter in vacuum, gas or liquid without touching the sample to provide resemblance of a micro-gravity environment. A sample trapped in AL in air provides exceptional spatial access to the sample. Therefore, AL enables a non-touching platform that technically permits non-touching manipulation by light, sound, electric field or magnetic field. While not yet established, embryos could in principle be levitated with no liquid immersion by using climate control (i.e. control on temperature, pressure and humidity) to avoid dehydration of the embryo. Such an application would enable wall-less and obstacle-less characterization and manipulation of the embryo with (i) lowered risk for contamination, (ii) no imaging artifacts arising from wave transmission through liquid or chamber walls and (iii) precise non-contacting manipulation since the transmitted waves traverse no walls. Studying an embryo with no surrounding medium would also limit the exchange of charges, magnetic domains, and molecules between the embryo and the surrounding immersion medium/chamber thus providing a new kind of a platform for characterization and manipulation experiments. Examples of other potential applications of AL of embryos are embryo/cell handling in embryonic cell transfer and *in vitro* fertilization.

In this study, zebrafish embryos were successfully levitated for up to 2000 s in an acoustic levitator. We observed no statistically significant levitation-induced death in embryos or abnormalities in their sound and pressure organs, *i.e.* otoliths or neuromasts. Based on the results AL may be a safe non-contacting and a non-destructive test platform to characterize or actuate zebrafish embryos.

## Methods

### Animals

We used wild-type animals of the Turku strain of zebrafish, *Danio rerio*. This strain has been maintained in the laboratory for over a decade and has been used in other studies^15^,^16^,^35^,^36^. Adult animals were bred and housed according to Westerfield^37^. The larvae were grown in embryo medium (E3, 5.00 mM NaCl, 0.44 mM CaCl2, 0.33 mM MgSO4 and 0.17 mM KCl), at 28 °C under a 14 h light and 10 h dark cycle. The experiments were done on animals younger than 5 dpf. The animal permit was granted from the Office of Regional Government of Southern Finland. All experiments were done in accordance with the relevant rules and regulations.

### Characterization of the levitating acoustic field

Scanned Laser-doppler vibrometry (LDV) (OFV-2570 controller, OVF-505 head; Polytec, Waldbronn, Germany; bandwidth 30 kHz – 24 MHz; velocity mode) was used to characterize the ultrasound field within the AL^38^,^39^,^40^. This approach permits characterizing the average pressure maxima along a path that is normal to the center axis of ultrasound transducer (Fig. 1A). This again permits estimating the pressure in the trapping node of the AL. Care was taken to ensure that the laser beam ran horizontally.

The laser rays (633 nm wavelength) travelled horizontally through the center axis of the cylinder symmetric AL (diameter of the levitator = 30 mm), reflect from a rigid reflector, and return along the same path back to the LDV (Fig. 1A). For each measurement point, the LDV detects an apparent change in optical path length (as a function of time) due to a pressure-induced alteration in the optical refractive index *n*. The change in *n* is caused by an acoustic pressure/density change along the optical path^41^. These pressure fluctuations in the standing wave inside the AL result from interference between vertical counter-travelling waves inside the trap. The signal representing the optical path as a function of time is recorded with an oscilloscope (Lecroy WaveSurfer 24Xs-A, Teledyne LeCroy Corp., Chestnut Ridge, NY, USA; 8 bit vertical resolution, 25 MHz sampling frequency, 300 averages). The measurement was repeated four times at each point. Using a custom-made software in Matlab (version 2012a; Mathworks Inc, Natick, MA), the recorded LDV signals were filtered with a 1000 point Savitzky-Golay low pass filter to remove noise. Following this, the maximum positive peak amplitude of the signals was determined. The pressure value in each scanned point was obtained from the apparent sound velocity values^40^ recorded by LDV as expresed by Eq. 1:


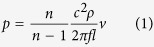


where *n* is the refractive index (1.000268533) calculated from the modified Edlén equation^42^,^43^,^44^ in standard laboratory conditions (50% RH, 23 °C, 101.325 kPa pressure), *c* is the speed of sound (345 m/s), *ρ* is the density of air (1.1919 kg/m^3^), *f* is the trapping frequency (35.2 kHz), *l* is the optical path length (30 mm) and *v* is the apparent velocity measured by LDV.

### Levitation experiments

Zebrafish embryos (2–14 hpf) were levitated at room temperature using a custom-made Langevin levitator^45^ with 11.61 ± 0.02 mm (mean ± S.D., *n *= 4) inter-reflector distance (Fig. 1) featuring two transducer elements (Pz26, Ø = 30 mm, thickness = 1 mm, Ferroperm Piezoceramics, Kvistgård, Denmark). The system was energized with a 35.2 kHz sinusoidal signal using a function generator (model 33120A, Hewlett Packard Inc., Palo Alto, CA, USA) and a power amplifier (model 500A100A, AR Inc., Souderton, PA, USA): The continuous-wave sinusoidal signal across each piezo element was 13.05 ± 0.06 Vpp (mean ± S.D., *n *= 5) or 13.26 ± 0.09 Vpp (mean ± S.D., *n *= 5) with or without a levitated droplet (ion-exchanged water), respectively.

**Figure 2:**
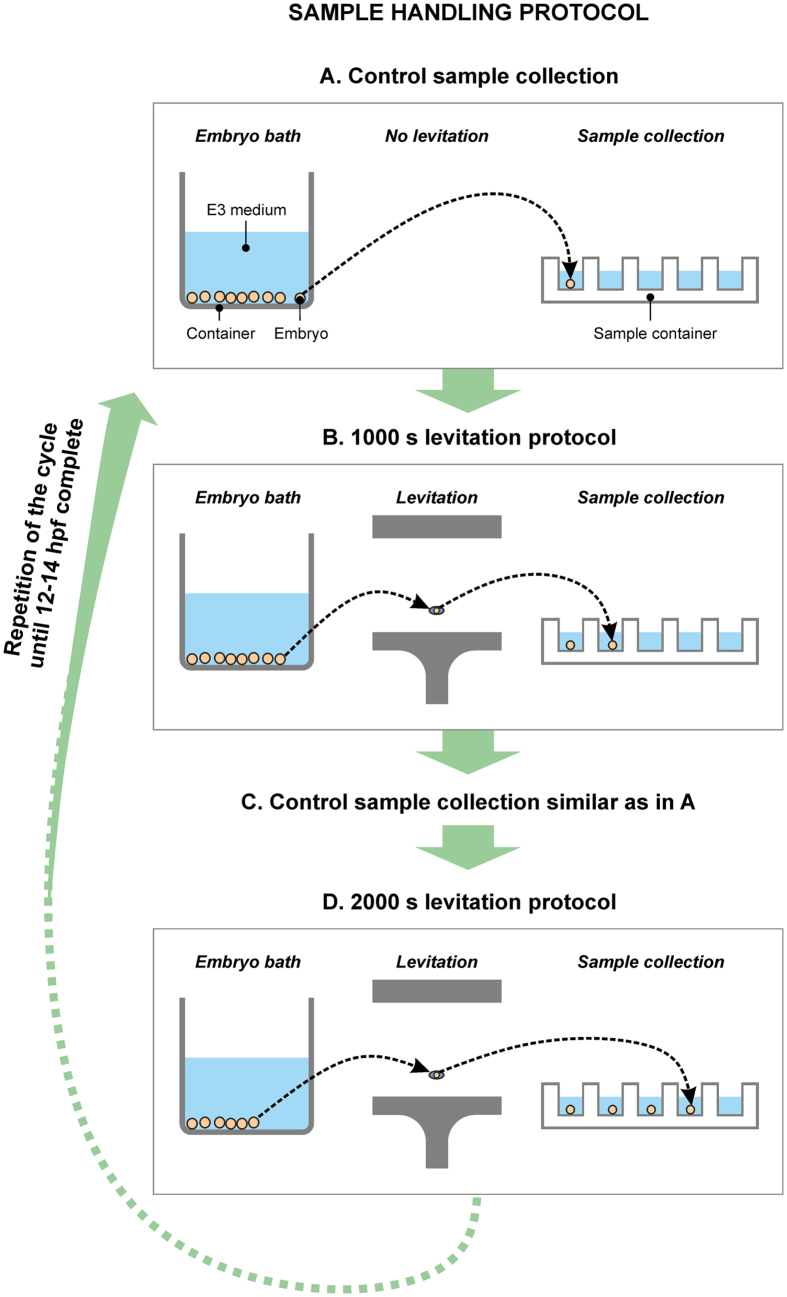
Levitation protocol. The zebrafish embryos were kept in a beaker containing E3 medium. Prior to levitation, one control sample was collected (**A**) and placed in a dish with small containers filled with 2 ml of E3 medium. This was followed by 1000 s levitation (**B**) after which the sample was collected and placed in the dish. Subsequently, a control sample was collected (**C**) similar as in A. After this 2000 s levitation was conducted (**D**) and the sample collected in a similar manner as in B. The cycle A–B–C–D was repeated until 12–14 hpf was complete.

**Figure 3:**
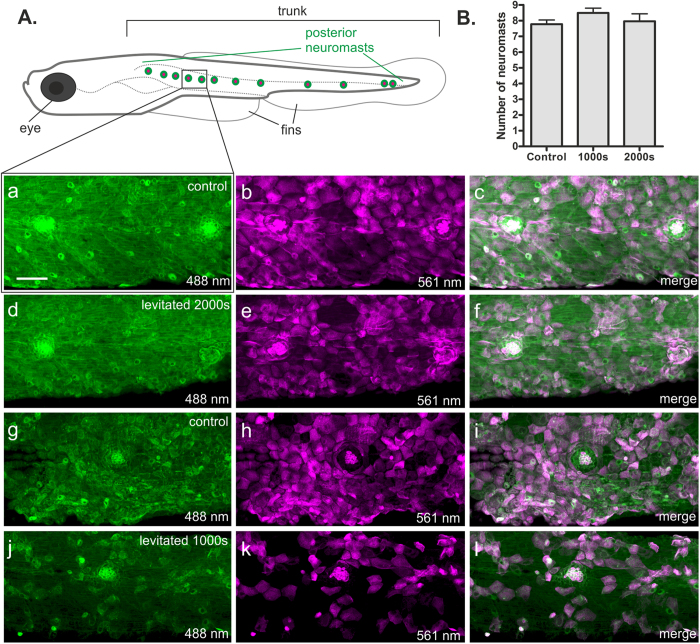
The morphology of some of the posterior lateral line neuromasts is visualized here. The schematic drawing of the lateral view of a zebrafish larva (**A**) shows the position of posterior neuromasts. One individual neuromast is depicted as a green circle with magenta filling, and the neuromasts are positioned along the lateral line of the zebrafish trunk. The neuromast is visualized in the merged images in white (c,f,i,l). These larvae were either levitated for 1000 s (*n *= 31, j–l), 2000 s (*n *= 27, d–f) or 0 s (*n *= 73, a–c, g–i). Anterior of the fish is oriented towards the left and the dorsal of the fish towards the top of the page. The number of neuromasts (mean ± SEM) did not differ between levitated and control larvae (**B**) (One-way analysis of variance, *p *> 0.05, followed by Tukey’s Multiple Comparison Test, *p *> 0.05, between all groups). Scale bar, 50 μm.

**Figure 4:**
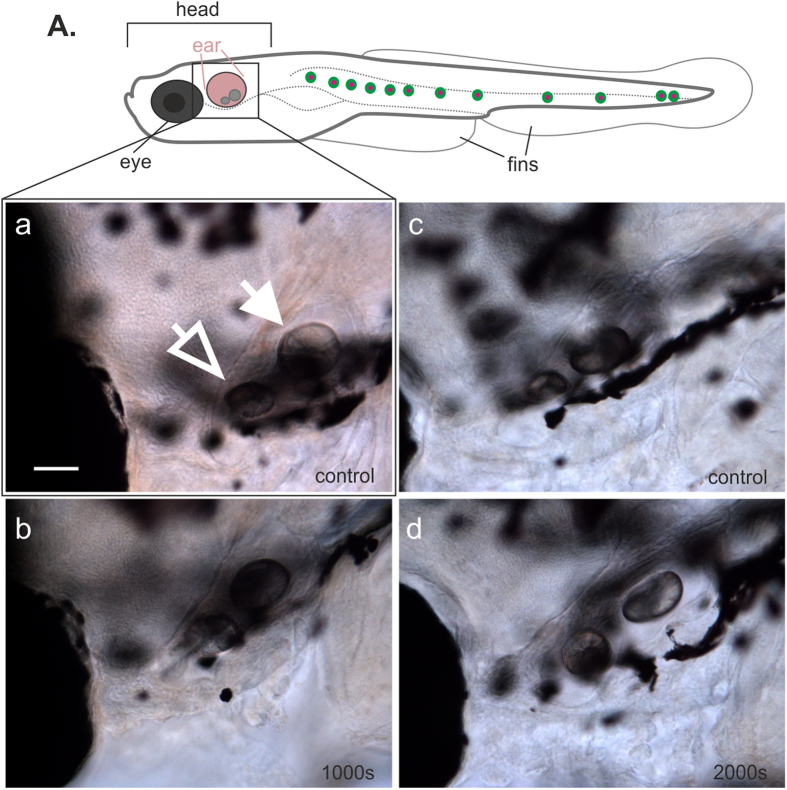
The development of otoliths in 5 dpf zebrafish larvae is visualized in this figure. The schematic drawing of the lateral view of the zebrafish larvae shows the position of the ear and its otoliths (**A**) depicted in pink. These larvae were levitated for 1000 s (*n *= 31) or 2000 s (*n *= 27). No difference in otolith placement, number or diameter (Student’s t-test, *p > *0.05) can be detected in either group when compared with control non-levitated animals (a–d). Open arrowhead indicates the smaller anterior otolith, and the closed arrowhead indicates the larger posterior otolith. Anterior of the fish is oriented towards the left and the dorsal of the fish towards the top of the page. Scale bar, 50 μm.

The embryos were inserted with a syringe and a metal needle and extracted from the trap using a disposable polymer pipette. The levitation protocol is demonstrated in Fig. 2. Following levitation the zebrafish were kept alive for 5 dpf.

### Lateral line

Neuromasts of the lateral line were labeled with the membrane stain FM^®^ 1–43FX (Invitrogen, Molecular probes, Eugene, OR, USA) and imaged using a transmission light microscope (see Imaging for details) as previously reported with minor modifications^24^. Briefly, 5 dpf animals were incubated in the staining solution for 10 minutes at room temperature (RT), rinsed with embryo medium, and fixed with 4% paraformaldehyde overnight at 4 °C. The next day the paraformaldehyde was removed, the animals were rinsed with phosphate buffered saline at RT, and infiltrated with 1:1 glycerol in phosphate buffered saline overnight at 4 °C. Larval fish were placed individually in a glycerol drop between cover glasses (24 × 60 mm, Microscope Cover Glasses, VWR, Radnor, PA, USA) that were attached together to form a sandwich by mounting smaller cover glasses (18 × 18 mm, Microscope Cover Glasses, VWR, Radnor, PA, USA) by silica grease (Baysilone-Paste, GE Bayer Silicones, Sigma-Aldrich Corporation, Saint Louis, MO, USA) to the ends of the larger cover glass.

### Imaging

Neuromasts of the lateral line were imaged with a Leica TCS SP2 confocal microscope (Leica Microsystems, Mannheim, Germany). Excitation wavelengths were 488 nm and 561 nm with the respective emission spectra of 500–550 nm and 600–650 nm. Sequential scanning was applied to avoid overlapping and bleed-through of the channels. Step size was set to 1 μm and each image was generated as an average of two frames in each focus plane. A Leica 506173 ∞ 0.17/D HCX PL APO 40X/0.75 U-V-I dry objective was used. Maximum projection images were created from the obtained image stacks with the conventional maximum projection algorithm by using the Leica confocal software (version 2.61, build 1537). Otoliths were imaged with an inverted Leica DM IRB microscope and a Leica 506503 ∞ 0.17/D HC PL FLUOTAR 20x/0.50 objective. Images were acquired with a Leica DFC 490 camera attached to the microscope and a Leica Application Suite version 2.8.1 analysis software (Leica Microsystems, Mannheim, Germany). Images were compiled into figure panels and analyzed in CorelDraw (Corel Corporation, Ottawa, Canada).

### Statistical analysis

To detect differences between groups Student’s t-test and one-way analysis of variance with Tukey’s Multiple Comparison Test was performed in GraphPad Prism (GraphPad Software, Inc., La Jolla, CA, USA). All statistical analyses were done both within the individual experiments, and on the merged data.

### Safety assessment

There are potential hazards since the E3 medium droplet dries with levitation time and since the ultrasound field may affect the embryo. We, therefore, run a separate series to define time points potentially critical for embryo safety. We levitated zebrafish embryos (*n *= 6 at *low humidity*, *i.e.* LH group; RH 13.3%, 22.8 °C and *n *= 6 at *medium-low humidity*, i.e. MLH group; RH 28.9%, 21.9 °C) in similar acoustic conditions as described earlier until (i) the embryo spontaneously exited the levitator or, if no spontaneous exit occurred, (ii) until the E3 had evaporated and embryo appears dry (qualitative assessment and extraction at time points 1, 1.25, 1.5 or 1.75 hrs from the beginning of levitation). We collected control embryos (*n *= 6 at *low humidity* and *n *= 6 at *medium-low* humidity) before each levitation event. All levitated and control embryos were imaged (Venus USB 2.0 camera, Modecom, Poland) before each levitation event and diameter *d*_[embryo]_ was determined as the average of the vertical and horizontal embryo diameter. The embryo volume *V*_[embryo]_ was determined assuming perfect sphericity: 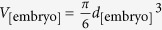. The levitated sample (contains E3 medium and the embryo) was imaged with light microscope (Stemi 2000-C stereo microscope, Oberkochen, Germany) and Thorlabs camera (0.2 frames per second, 1.3 megapixels, model: DCC1645C-HQ, Newton, NJ) throughout the levitation event. The width 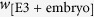 and height 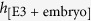 of the levitated sample were determined from the acquired images using a custom-made Matlab program (version R2012a, Mathworks Inc, Natick, MA, USA). The sample was separated from the background by thresholding (pixel values greater than a manually defined threshold value represent the sample). Maximum horizontal or vertical distance of pixel (pixel side length 5.8 μm) belonging to the sample represented the width and the height of the sample, respectively. The sample volume was determined as: 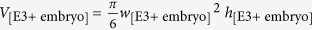. Finally, four time points relevant for embryo safety were determined: (i) time (*t*_1_) when 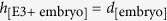 (represents the circumstance in which the embryo is potentially exposed to direct air contact); (ii) time (*t*_2_) when 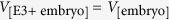 (represents the circumstance in which the E3 medium has dried and the entire embryo is potentially exposed to direct air contact); (iii) time when embryo spontaneously exits the levitator (*t*_3_); and (iv) time (*t*_4_) when embryo appears to have dried and is extracted from the levitator. The mortality rate was determined at 12–14 hpf.

## Additional Information

**How to cite this article**: Sundvik, M. *et al.* Effects of acoustic levitation on the development of zebrafish, *Danio rerio*, embryos. *Sci. Rep.* **5**, 13596; doi: 10.1038/srep13596 (2015).

## Figures and Tables

**Table 1:** Statistical analysis of death rate in levitated and control zebrafish.

Tukey’s Multiple Comparison Test	Mean Diff.	P value	95% Cl of diff
Control vs 1000 s	1.667	P > 0.05	−7.669 to 11.00
Control vs 2000 s	0.3333	P > 0.05	−9.003 to 9.669
1000s vs 2000 s	−1.333	P > 0.05	−10.97 to 8.003

**Table 2:** Statistical analysis of neuromast number in levitated and control zebrafish.

Tukey’s Multiple Comparison Test	Mean Diff.	P value	95% Cl of diff
Control vs 1000 s	0.1692	P > 0.05	−1.700 to 2.039
Control vs 2000 s	0.3526	P > 0.05	−1.401 to 2.106
1000 s vs 2000 s	0.1833	P > 0.05	−1.968 to 2.334

**Table 3:** Parameters and critical time points for safety assessment.

Parameter	Low humidity (LH) group	Medium-low humidity (MLH) group
Humidity (RH %)	13.3	28.9
Room temperature (°C)	22.8	21.9
Number of levitated samples (unless specified)	6	6
Initial sample volume (E3 droplet + embryo) (μL)	18.0 ± 4.3	24.2 ± 3.3
Initial embryo volume (μL)	0.9 ± 0.1	0.9 ± 0.0
Mortality at 12–14 hpf	100	100 (*n *= 5)
Time (*t*_1_) when sample height (E3 droplet + embryo) = embryo diameter (s)	2056 ± 667	3359 ± 793
Time (*t*_2_) when sample volume (E3 droplet + embryo) = embryo volume (s)	2990 ± 375	4985 ± 482
Time (*t*_3_) when embryo spontaneously exited the levitator (s)	3428 ± 18 (*n *= 2)	4735 (*n *= 1)
Time (*t*_4_) when embryo dried and was manually extracted from levitator (s)	3569 ± 19 (*n *= 4)	5578 ± 391 (*n *= 5)
Samples failures at insertion	1	0
Samples failures at extraction	0	1

All safety time points exceeded the levitation times 1000 s and 2000 s applied in study. No control fish (*n *= 12) were found dead at mortality assessment (12–14 hpf), whereas all fish (*n *= 11) were dead in the levitation group after 3428–5578 s levitation.
